# Lutembacher’s Syndrome Associated with Meningioma

**DOI:** 10.5812/cardiovascmed.6557

**Published:** 2013-02-24

**Authors:** Mohammadsaeid Ghiasi, Alireza Jalali, Hamidreza Taghipour, Shiva Khaleghparast, Hamid Mohamadpour, Behshid Ghadrdoost

**Affiliations:** 1 Department of Anesthesiology, Baqiyatallah University of Medical Sciences, Tehran, IR Iran; 2 Rajaie Cardiovascular Medical and Research Center, Tehran University of Medical Sciences, Tehran, IR Iran; 3 Department of Radiology, Shahid Beheshti University of Medical Science, Tehran, IR Iran

**Keywords:** Heart Septal Defects, Atrial, Mitral Valve Stenosis, Meningioma

## Abstract

A 49-year-old man with Lutembacher's syndrome associated with frontal meningioma referred to our hospital. He also suffered from exertional dyspnea. Transthoracic echocardiography demonstrated mitral valve area of 1.48 cm2, moderate mitral stenosis, and left atrial dimension (LAD) of 5.6 cm with no clot. TEE revealed severe mitral stenosis, mitral valve area of 1.05 cm2 with wilkins 8-10 score, ejection fraction of 50%, and enlarged left atrium (LAD = 5.8 cm) with no clot. Induction of anesthesia was commenced taking into account the patient’s specific circumstances, which meant the risk of surgery was high. During surgery, the mitral valve was replaced and the atrial septal defect was repaired without a patch. This case underscores the significance of the adoption of an appropriate therapeutic strategy in the treatment of Lutembacher's syndrome with meningioma before meningioma surgery.

## 1. Introduction

Lutembacher's syndrome is a type of congenital cardiac abnormality comprised of atrial septal defect (ASD) and mitral stenosis (MS) (1-5). It is vitally important that an accurate diagnosis be made so as to ensure optimal surgical correction (4) or transcatheter therapy (4,5) of the syndrome in adults (1). The incidence of MS in patients with ASD is 4% and the incidence of ASD in patients with MS is 0.6%-0.7% (6). Of the different diagnostic modalities available in the armamentarium for Lutembacher's syndrome, echocardiography seems to be the most efficient (7). 

Incidence of meningiomas is 13% to 26% in intracranial tumors. Meningiomas, albeit benign in 90% of cases (8), can affect many parts of the brain with varying symptoms such as headache, vertigo, and seizure, depending on where the tumor develops (9). This case report describes a patient with severe MS and ASD associated with frontal meningioma. To our knowledge, this combination has never been reported in the literature.

## 2. Case Report

**Figure 1.**
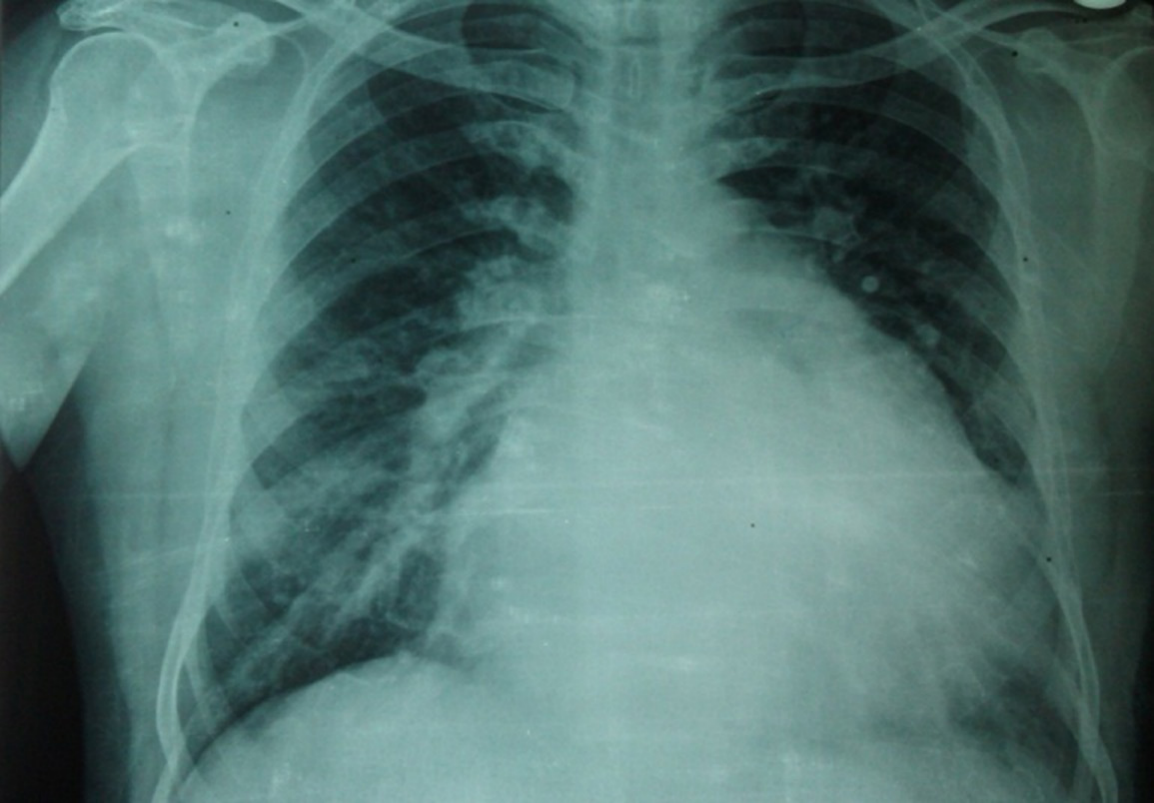
Chest x-ray shows pulmonary overflow, left atrial enlargement, and cardiomegaly.

A 49-year-old man was admitted to our hospital on 11 Aug. 2011. He suffered from exertional dyspnea of 3 months’ duration and with gradual intensification. Physical examination showed blood pressure on admission of 140/85 mmHg, pulse of 120 bpm, respiratory rate of 16 breaths per minute, and grade 2/6 diastolic murmur in the mitral valve. ECG demonstrated atrial fibrillation with appropriate ventricular response (120 bpm). His body weight is 74 kg. Left atrium (LA) enlargement as well as cardiomegaly was evident in chest X-ray. Pulmonary overflow is also observed in chest X-ray (Figure1). Routine hematologic, biochemical, and rheumatic serologies were within normal ranges.

**Figure 2.**
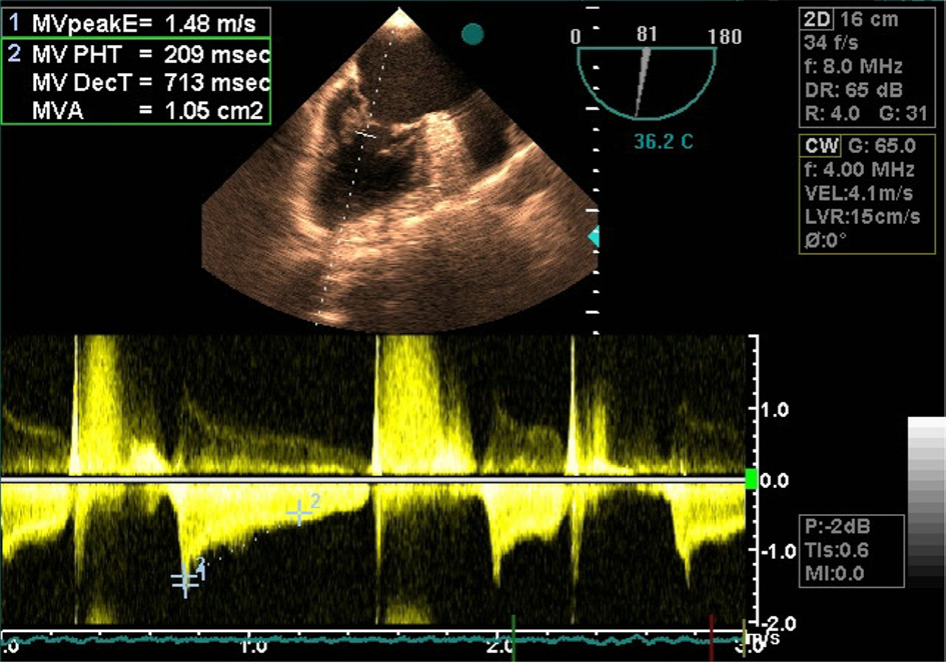
Transesophageal echo before surgery

**Figure 3.**
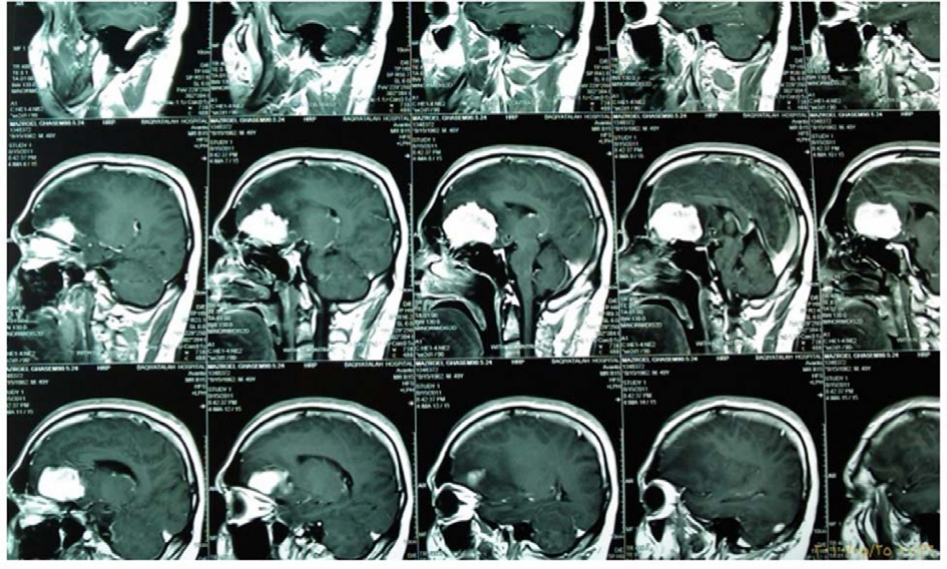
Brain magnetic resonance imaging shows frontal lobe meningioma

Transthoracic echocardiography (TEE) demonstrated mitral valve area of 1.48 cm^2^(moderate MS) normal LV size with ejection fraction of 50%, and left atrial dimension (LAD) of 5.6 cm with no clot. TEE revealed severe MS, mitral valve of 1.05 cm^2^with wilkins 8-10 score, ejection fraction of 50%, enlarged LA (LAD = 5.8 cm) with no clot, and aneurysmal IAS with secundum ASD of 10mm (Figure 2). Color flow doppler by TEE showed left-to-right shunt at the atrial septum. Additionally, there was moderate tricuspid regurgitation with SPAP of 40 mmHg. Given that the patient had experienced seizure one year previously, he was subjected to full evaluation and diagnosed with frontal meningioma (Figure 3). Brain magnetic resonance imaging showed a 55×50 mm mass lesion in the anterior mid-sagittal fossa, consistent with district meningioma as well as a heterogeneously limited district with edema and compression to the adjacent area. The gray and white matters had a normal appearance and signal (Figures 3).

A neurosurgeon was consulted and decision was made to perform brain surgery after cardiac surgery. Because the patient’s intracranial pressure was high, 8mg Dexamethasone (IV, TDS) was administered to the patient a few days before the operation and was continued up to a few days after the operation. Also, 300 mg Phenytoin capsules (TDS) were administered one week before surgery for up to three days thereafter. The night before surgery, the patient was alert and answered all the questions. The ECG demonstrated atrial fibrillation, but drug premedication was not considered. The following morning, the patient was transferred to the operating room, where the bed was set up at 30 degrees. ECG and pulse oxymetry monitoring were conducted, and local anesthesia was commenced subcutaneous then IV line catheter and arterial catheter inserted and blood pressure directly monitored. The cardiac rhythm was still atrial fibrillation with blood pressure of 110/80 and heart rate of 80-90 bpm. At this point, 20mg Furosemide was administered intravenously followed by the infusion of 300 ml Mannitol 20%. Anesthesia was induced subsequently as follows:

First, 100 mg IV Xylocaine and then 7.5mg Diazepam were injected to induce sleepiness. Next, 8 mg Pancuronium was injected and the patient was ventilated with a mask. Afterward, 500 micro/ kg Fentanyl was given following a period of full relaxation and for 3 minutes ventilated. Before intubation, 250 mg Sodium Thiopental was intravenously administered. The patient was intubated easily and ventilated, with the ventilator being set in such a way that it could maintain ETCO2 at about 25-30 mmHg by capnography. The transesophageal probe was thereafter inserted. Additionally, 15 mg Midazolam, 150 micro/kg Fentanyl, and 150 mg Atracurium were used for the maintenance of surgery duration.

During surgery, systolic blood pressure were maintained at about 95-125mmHg and mean arterial pressure of 80-90mmHg, during cardiopulmonary pump (CPB). The patient was ultra-filtered during CPB and excreted about 1500 ml fluid. After the establishment of CPB, the mitral valve was replaced with a metallic No.31 St Jude valve and the ASD was repaired without a patch. The heart beat was re-commenced, and cardiac cavities conditions were regularly monitored. Finally, the patient was separated from the CPB in sinus rhythm. The function of mechanical mitral prosthesis was assessed by TEE and there was acceptable prosthesis function (meanPG = 2.14 mmHg, peakPG= 4.25, MV VTI = 20.22 cm). There was not significant paravalvular leakage. Also, the patient’s ASD was evaluated via TEE and there was no defect. Intraoperative urine volume was about 1200 ml. At the end of the surgical operation, no blood products were administered to the patient, and he was transferred to the ICU in sinus rhythm and with a blood pressure of 110/80 mmHg.

The following measures were taken in the ICU:

1. Head-up position about 30 degrees,
2. Regulating the TV and RR of the ventilator to maintain Pa CO2 at 25- 30 mmHg,
3. Controlling and maintaining the blood pressure at about 100-120 mmHg,
4. Injecting 5 mg Methadone intramuscularly on arrival,
5. Administrating 8mg Dexamethasone intravenously once every 8 hours,
6. Infusing Mannitol 20%, 100 ml once every 6 hours and then gradually discontinuing it over a 48-hour period, and
7. Administrating 300 mg Phenytoin capsules once every 8 hours after the patient had resumed eating by mouth.

Approximately, 4 hours after arrival in the ICU, the patient awakened. By taking the patient’s hemodynamic stability, lack of drainage, and partial consciousness into account, the process of weaning was started, and the patient was extubated about an hour later. The patient’s awareness and orientation was adequate, and sense and movement of his limbs were acceptable.

## 3. Discussion

In the patient presented here, MS was diagnosed based on the mitral valve morphology and sensation thereof. It has been suggested that some of the changes noted with respect to the mitral valve in Lutembacher’s syndrome are associated with ASD. The changes in the mitral valve leaflets in our case were stenotic. The presence of increased pulmonary blood flow and significant transmitral pressure gradient denotes severe MS.

Meningiomas are usually benign but can show another form in 1-10% of cases in different series (10). In recent years, there have been several case reports of ASD associated with MS; none of them, however, have reported it with meningiomas. In 1995, a 63-year-old woman with mitral valve area of 1.48 cm2 and mild mitral regurgitation associated with a partial type of common atrioventricular canal was presented (11). Our patient had severe MS with a mitral valve area of 1.1 cm2 with score 8-10. Another case report in 1953 introduced a 30-year-old man with Lutembacher’s syndrome treated successfully via mitral commissurotomy because surgical risk was high. Prior to the onset of his disease, the patient in that report did not have orthopneic or nocturnal dyspnea; however, when he became ill, he developed both (12). Our patient had exertional dyspnea, as well. It should be noted that whereas the patient in the case report above had grade II diastolic and grade III systolic murmurs, our patient had grade II/VI diastolic murmur. Overall, the 30-year-old patient’s condition marked enlargement of the right ventricle, left auricle, and pulmonary vessels, which complexed him and prompted the decision for his commissurotomy (12). Concurrency of meningioma with Lutembacher’s syndrome is extremely rare. The present case highlights the significance of the selection of an appropriate therapeutic strategy for the treatment of this syndrome prior to meningioma surgery.
